# Coxsackievirus A6 Induces Necroptosis for Viral Production

**DOI:** 10.3389/fmicb.2020.00042

**Published:** 2020-02-04

**Authors:** Shuxia Zhang, Xiaoyan Yu, Xiangling Meng, Wenbo Huo, Ying Su, Jinming Liu, Yumeng Liu, Jun Zhang, Shaohua Wang, Jinghua Yu

**Affiliations:** ^1^Department of Experimental Pharmacology and Toxicology, School of Pharmaceutical Science, Jilin University, Changchun, China; ^2^Institute of Virology and AIDS Research, The First Hospital of Jilin University, Jilin University, Changchun, China

**Keywords:** CA6, necroptosis, viral production, RIPK3, host–pathogen interaction

## Abstract

Hand, foot, and mouth disease (HFMD) is a febrile exanthematous disease with typical or atypical symptoms. Typical HFMD is usually caused by enterovirus 71 (EV71) or coxsackievirus A16, while atypical HFMD is usually caused by coxsackievirus A6 (CA6). In recent years, worldwide outbreaks of CA6-associated HFMD have dramatically increased, although the pathogenic mechanism of CA6 is still unclear. EV71 has been established to induce caspase-dependent apoptosis, but in this study, we demonstrate that CA6 infection promotes a distinct pathway of cell death that involves loss of cell membrane integrity. Necrostatin-1, an inhibitor of necroptosis, blocks the cell death induced by CA6 infection, but Z-DEVD-FMK, an inhibitor of caspase-3, has no effect on CA6-induced cell death. Furthermore, CA6 infection up-regulates the expression of the necroptosis signaling molecule RIPK3. Importantly, necrostatin-1 inhibits CA6 viral production, as assessed by its ability to inhibit levels of VP1 protein and genomic RNA and infectious particles. CA6-induced necroptosis is not dependent on the generation of reactive oxygen species; however, viral 3D protein can directly bind RIPK3, which is suggestive of a direct mechanism of necroptosis induction. Therefore, these results indicate that CA6 induces a mechanism of RIPK3-dependent necroptosis for viral production that is distinct from the mechanism of apoptosis induced by typical HFMD viruses.

## Introduction

Hand, food, and mouth disease (HFMD) is a highly contagious disease caused by enterovirus infection. It especially poses a health problem in young children under 5 years of age worldwide. The most common causative agents of typical HFMD are enterovirus 71 (EV71) and coxsackievirus A 16 (CA16), though coxsackievirus 6 (CA6) has emerged as a causative agent of an atypical form of the disease. In recent years, outbreaks of CA6-associated HFMD have markedly increased worldwide, and especially in China (Gao et al., 2016; Laga et al., 2016; Li J.S. et al., 2016; Li W. et al., 2016; Mirand et al., 2016; Anh et al., 2018; Wang S.H. et al., 2018; Du et al., 2019). Although vaccines and drug have been developed for HFMD, they principally target EV71 and CA16; strategies for preventing and treating CA6-related-HFMD are scarce, and the current understanding of the pathogenic mechanism of CA6 remains limited.

The typical symptoms of HFMD caused by EV71 or CA16 include flat discolored spots or bumps and sometime, heart inflammation, brain inflammation, or acute flaccid paralysis (Chan et al., 2003; Wang et al., 2012); however, CA6 causes atypical HFMD, characterized by severe rash, onychomadesis in young children, and a higher rate of infection in adults. Furthermore, the pathogenic mechanism of EV71 has been heavily investigated. For example, EV71 has been shown to induce apoptosis that is characterized by cell shrinkage, nuclear condensation, and plasma membrane blebbing in different cell lines (Chang et al., 2004; Lu et al., 2013; Song et al., 2018). EV71-associated apoptosis has also been shown to involve the activation of caspases during viral replication (Chang et al., 2004). Indeed, many viruses induce apoptosis, including but not limited to CA16 (Li et al., 2014), human immunodeficiency virus HIV-1 (Westendorp et al., 1995), hepatitis C virus (Zhu et al., 1998), Epstein–Barr virus (Le Clorennec et al., 2006), and human papillomavirus (Wang et al., 2011). Therefore, apoptosis represents the dominant mechanism by which virus-infected cells undergo programmed cell death. Nevertheless, it remains unknown whether CA6 induces apoptosis.

Coxsackievirus 6 belongs to the *Enterovirus* genus of the Picornaviridae family, which has a single-stranded, positive-sense RNA genome of about 7400 bp that could encode a poly-protein (about 2200 amino acids). In host cells, this poly-protein is further cleaved into four structural (VP1 to VP4) and seven non-structural (2A to 3D) proteins by viral non-structural 2A and 3C proteases (Solomon et al., 2010). Besides 2A and 3C proteins, non-structural 3D protein as an RNA-dependent RNA polymerase is responsible for viral genome replication via incorporating nucleotides into RNA strand (Baltimore, 1964). Recently our studies have demonstrated that 3C and 3D exert other roles that affect the life cycle of the virus (Song et al., 2018; Wang Z. et al., 2018).

As an alternative to apoptosis, necroptosis is an inflammatory type of cell death characterized with cell swelling, loss of plasma membrane integrity, and release of cytosolic contents into the extracellular space (Orzalli and Kagan, 2017). Unlike apoptosis, necroptosis is independent of caspase activity. Generally, when membrane receptors or cytosolic receptors are activated, RIPK1 (receptor-interacting protein kinase 1) associates with RIPK3 via the RIP homotypic interaction motif domain, and this interaction promotes RIPK3-mediated phosphorylation of RIPK1, which then phosphorylates RIPK3. Activated RIPK3 recruits and phosphorylates MLKL, resulting in cell lysis (Cho et al., 2009; He et al., 2009), or activates Ca^2+^-calmodulin-dependent protein kinase (CaMKII) to induce necroptosis (Zhang et al., 2016). RIPK3-dependent necroptosis has been demonstrated to be activated in response to bovine parvovirus (BPV) (Abdel-Latif et al., 2006), murine cytomegalovirus (Upton et al., 2012), Influenza A virus (Thapa et al., 2016), and reovirus (Berger et al., 2017). Furthermore, high amounts of reactive oxygen species (ROS) have been shown to induce necroptosis (Takemoto et al., 2014; Schenk and Fulda, 2015; Muraro, 2018).

In this study, we demonstrate that the CA6-induced cell death mechanism is distinct from the apoptotic mechanism of EV71 and is instead characterized by RIPK3-dependent necroptosis. CA6-induced necroptosis is not dependent on the generation of ROS, although viral 3D protein can directly bind to RIPK3. Importantly, necrostatin-1 inhibits necroptosis and viral production. These results further advance our understanding of the pathogenic mechanisms of CA6 and provide a potential target for the treatment and prevention of HFMD.

## Materials and Methods

### Cells and Viruses

Human rhabdomyosarcoma RD cells (No CCL-136), human embryonic kidney cells (HEK 293T cells) (No CRL-11268), and human cervix epithelial HeLa (No CCL-2) were purchased from the ATCC (Manassas, VA, United States). Cells were grown in Dulbecco’s Modified Eagle’s Medium (DMEM, Hyclone, Logan, UT, United States) supplemented with 10% fetal bovine serum (FBS, GIBCO BRL, Grand Island, NY, United States). The 98 strain of CA6^1^ was provided by the Jilin Provincial Center for Diseases Control and Prevention (Changchun, China). Viruses were propagated in RD cells, and the supernatants were collected and stored at −80°C as our previous studies (Yu et al., 2015; Wang et al., 2017).

### Viral Titer Determination

As previous study (Zhong et al., 2017), the viral titer in a microtitration assay was determined by measuring the TCID_50_ in RD cells. Virus was serially prepared after 10-fold dilution, and 100 μL virus/well was inoculated in octuplicate in 96-well plates. The cytopathic effect was measured once per day until the experimental endpoint was reached. The TCID_50_ was determined according to the Reed–Muench method (Reed and Muench, 1983) based on that viruses of 1 × 10^5^ TCID_50_/mL will produce 0.7 × 10^5^ plaque forming units/mL as our previous studies (Yu et al., 2015; Wang et al., 2017).

### Propidium Iodide Staining

After gently removing the culture medium, RD cells were stained *in situ* with 4.5 μM of propidium iodide (PI) (40747-B, Yeasen, Shanghai, China) in buffer (40747-C, Yeasen, Shanghai, China) for 15 min at room temperature in the dark place. The morphological changes were observed and photographed by optical microscopy (Olympus, Tokyo, Japan). PI staining was observed at excitation wavelength 488 nm with emission filter 630 nm by fluorescence microscopy (Leica, Nussloch, Germany).

### Infection

Cells were mock-infected or infected with CA6 at an MOI of 5, or EV71 at an MOI of 1. After 2 h of virus absorption, cells were washed once with PBS, and cells were cultured with fresh culture medium or drug treated medium.

### Drugs Treatment

After 2 h of virus absorption, cells were washed once with PBS, and cells were cultured with different dose (100, 150, and 200 μM) of necrostatin-1 (A4213, ApexBio, United States), different dose (100, 180, and 260 μM) of necrostatin-2 (A3652, ApexBio, United States) or different dose (1, 2, and 4 mM) of *N*-acetyl-L-cysteine (NAC, A9165, Sigma, St. Louis, MO, United States) for determination of applied concentration. And 20 μM of Z-DEVD-FMK (A1920, ApexBio, United States) as apoptosis inhibitor, 150 μM of necrostatin-1 as necroptosis inhibitor, 180 μM of necrostatin-2 as necroptosis inhibitor, or 2 mM of NAC as ROS scavengers was treated at 2 h post infection of CA6 or EV71.

### Cell Counting Using a Hemocytometer

Trypan Blue (Sigma, St. Louis, MO, United States) was used as a vital stain. Live cells appeared colorless and bright (refractile) under phase contrast, while dead cells stained blue and were non-refractile. After staining with a final concentration 0.2% trypan blue, live cells were visualized and counted using a hemocytometer.

### Western Blot Analysis

As our previous studies (Yu et al., 2015; Wang et al., 2017), virus-infected and mock-infected cells and/or together with drug treatment were collected at indicated times. Cells were lysed directly in sodium dodecyl sulfate (SDS) sample buffer [60 mM Tris–HCl (pH 6.8), 2% SDS, 10% glycerol, 5% 2-mercaptoethanol, 0.01% bromophenol blue], followed by boiling for 10 min. Whole-cell lysates were further subjected to SDS–PAGE. Proteins were transferred to nitrocellulose membranes (Bio-Rad) and detected with corresponding primary and alkaline phosphatase-conjugated secondary antibody. The membranes were then reacted with 5-bromo-4-chloro-39-indolylphosphate (BCIP) and nitro-blue tetrazolium (NBT) substrate (Sigma, St. Louis, MO, United States). The following antibodies were used: anti-RIPK3 (17563-1-AP, Proteintech), anti-p-MLKL (#91689, Cell Signal), anti-MLKL (#14993, Cell Signal), anti-VP1 (GTX132346, Genetex), anti-histone and anti-HA (GenScript), and anti-tubulin (11224-1-AP, Proteintech). Anti-mouse or rabbit secondary antibodies from goat were obtained from Jackson ImmunoResearch.

### Immunofluorescence Staining

RD cells (1 × 10^6^) were cultured in 6 cm culture dishes for 24 h and then were mock-infected or infected with CA6 (MOI = 5) for 36 h. At 36 h after post-infection, cells were fixed with 4% paraformaldehyde for 15 min. The cells were then rinsed three times with 1× PBS (5 min per wash) and blocked for 60 min in blocking buffer (1× PBS/5% normal serum/0.3% Triton X-100) at room temperature. The cells were then incubated with mouse anti-RIPK3 (17563-1-AP, Proteintech) antibody or together with HA antibody (715500, Invitrogen, United States) overnight at 4°C. After three washes in PBS, samples were incubated with FITC-goat anti-Mouse IgG (H + L) (Sungene Biotech, China), or CoraLite 594-conjugated Goat Anti-Mouse IgG (H + L) (Proteintech, China), or FITC-goat anti-Rabbit IgG (H + L) (Sungene Biotech, China) for another 2 h at room temperature. Cells were counterstained with Hoechst 33258 (Sigma, St. Louis, MO, United States) to visualize nuclear DNA on fluorescence microscopy at excitation wavelength 350 nm with emission filter 460 nm (Leica, Nussloch, Germany).

### Quantitative Real-Time RT-PCR

According to our previous studies (Yu et al., 2015; Wang et al., 2017), intracellular viral genome RNA was detected through targeting VP1 primers, and GAPDH was chose as internalization control. VP1 forward primer: AATGAGGCGAGTGTGGAAC; VP1 reverse primer: AGGTTGGACACAAAAGTGAACT; GAPDH forward primer: GCAAATTCCATGGCACCGT; GAPDH reverse primer: TCGCCCCACTTGATTTTGG. The fold changes were calculated relative to GAPDH using the 2^–ΔΔCt^ method for VP1, and ΔΔCt = [(VP1-GAPDH) of drug treatment − (VP1-GAPDH) of control treatment].

### Flow Cytometric Analysis by the 2′,7′-Dichlorofluorescin Diacetate Staining

Mock infected or CA6 infected RD cells were trypsinized and collected. Then the cells were stained with 10 μM of 2′,7′-dichlorofluorescin diacetate (DCF-DA) in DMEM with 10% FBS at 37°C for 60 min in the dark. After incubation, the cells were washed once with PBS. Mock infected RD cells are designed as negative control. The samples were analyzed by flow cytometry at excitation wavelength 480 nm with emission filter 525 nm (Yang et al., 2002).

### Plasmid Transfection

According to our previous studies (Yu et al., 2015; Wang et al., 2017), 2 μg of VR1012-HA plasmid, VR1012-3C-HA plasmid, or VR1012-3D-HA plasmid with 6 μL of Lipofectamine 2000 (Invitrogen, United States) were transfected into cells in 3.5-cm cell culture dishes. We replenished the empty plasmid VR1012 to maintain a final concentration of 2 μg plasmid/well for dose-dependent test of 3D. Amounts of plasmid and Lipofectamine 2000 were increased for other experiments.

### Immunoprecipitation

293T cells (5 × 105 cells/well) were cultured in 10 cm dishes for 24 h and then were mock-transfected or transfected with 10 μg of VR1012-HA, VR1012-3D-HA, or VR1012-3C-HA for 36 h. Then the cells were harvested in IP-lysis buffer [50 mM Tris (pH 7.4), 150 mM NaCl, 1% NP-40, 0.25% sodium deoxycholate, 1 mM EDTA], and the lysates were added to mouse anti-RIPK3 (sc-374639, Santa Cruz, United States) antibody for immunoprecipitation of RIPK3 in HA-, 3C-HA-, and 3D-HA-expressed cells. The bound proteins were analyzed by Western blotting by RIPK3 (17563-1-AP, Proteintech) and HA (715500, Invitrogen, United States) antibody.

### Statistical Analyses

Statistical differences were analyzed using the Student’s *t*-test. Multiple group differences were assessed using one-way ANOVA in SPSS 10.0. Data are presented as means and standard deviations (SD). *P-values of <0.05 were considered statistically significant.

## Results

### CA6 Infection Induces Cytopathic Effects, Though the Morphology After CA6 and EV71 Infection Is Different

**FIGURE 1:**
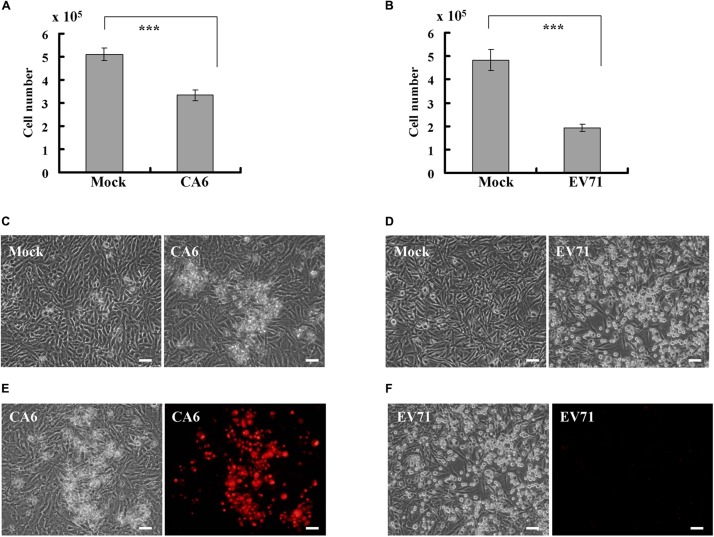
CA6 induces cytopathic effects in RD cells. **(A)** Cell number analysis after CA6 infection (MOI = 5) at 36 h. The cell numbers were counted by trypan blue staining. The results represent the mean ± SD of three independent experiments. ***P < 0.001. **(B)** Cell number analysis after EV71 infection (MOI = 1) at 24 h. The cell numbers were counted by trypan blue staining. The results represent the mean ± SD of three independent experiments. ***P < 0.001. **(C)** Cell morphologic analysis after mock or CA6 (MOI = 5) infection was performed at 36 h post infection. Results are representative of three independent experiments. Bar = 20 μm. **(D)** Cell morphologic analysis after mock or EV71 (MOI = 1) infection was performed at 24 h post infection. Results are representative of three independent experiments. Bar = 20 μm. **(E)** Cell morphologic analysis with propidium iodide staining after CA6 (MOI = 5) infection was performed at 36 h post infection by light microscopy (left) or fluorescence microscopy (right). Results are representative of three independent experiments. Bar = 20 μm. **(F)** Cell morphologic analysis with propidium iodide staining after EV71 (MOI = 1) infection was performed at 24 h post infection and assessed by light microscopy (left) or fluorescence microscopy (right). Results are representative of three independent experiments. Bar = 20 μm.

Given that EV71, a causative agent for typical HFMD, is known to induce an obvious cytopathic effect in RD cells (Chang et al., 2004; Lu et al., 2013; Song et al., 2018), we sought to determine whether CA6, a causative agent for atypical HFMD, also induces a cytopathic effect. At 24 h post infection, CA6 did not cause a statistical decrease in the cell number (data not shown); however, at 36 h post infection, CA6 decreased the cell number from 5.1 ± 0.26 × 10^5^ to 3.33 ± 0.23 × 10^5^ (Figure 1A). By comparison, at 24 h post infection, EV71 decreased the cell number from 4.83 ± 0.45 × 10^5^ to 1.93 ± 0.15 × 10^5^ (Figure 1B). These results suggest that CA6 can induce a cytopathic effect, although the time course for CA6-mediated cytopathy is extended relative to the time course for EV71-mediated cytopathy.

To determine whether the cytopathic effect caused by CA6 at 36 h post infection is similar to the cytopathic effect caused by EV71 at 24 h post infection, we performed morphology analysis. At 36 h after CA6 infection, many cells were floating, and others appeared ruptured (Figure 1C). In contrast, at 24 h after EV71 infection, the cells were condensed, rounded up, and detached from the bottom of the dish (Figure 1D). Therefore, although both viruses induced cytopathy, the mechanism of cell death induction by CA6 and EV71 appeared to differ.

To further distinguish the cell death induced by CA6 infection and EV71 infection, we utilized PI to assess plasma membrane rupture. After CA6 infection, the dying cells showed signs of cellular membrane permeability (Figure 1E), which is a mark of necroptosis (Abdel-Latif et al., 2006; Cho et al., 2009; He et al., 2009). In contrast, cellular PI staining was not observed after EV71 infection (Figure 1F), which is more suggestive of apoptosis and is consistent with the findings of previous studies (Chang et al., 2004; Song et al., 2018). Therefore, these results raise the possibility that CA6 might induce cytopathic effects through necroptosis, rather than apoptosis.

### CA6 Infection Induces a Necroptotic Form of Cell Death

**FIGURE 2:**
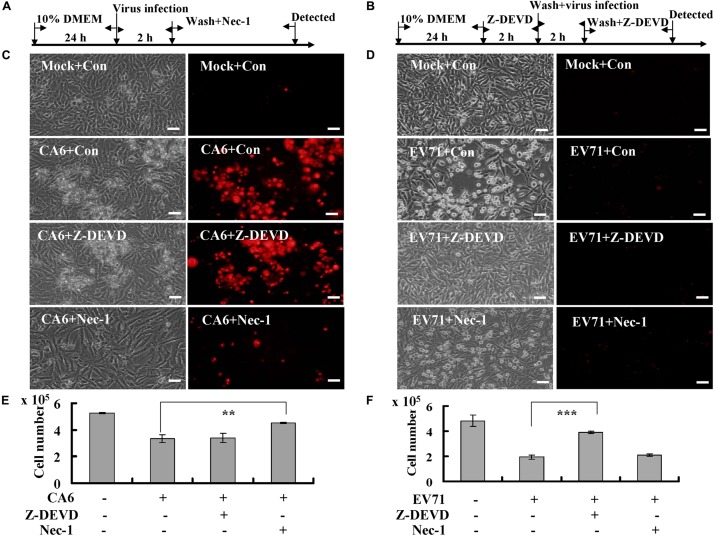
Necrostatin-1 inhibits cell death induced by CA6 infection. **(A)** Flow diagram of RD cells treated with necroptosis inhibitor (necrostatin-1). RD cells were mock-infected or infected with CA6 (MOI = 5) or EV71 (MOI = 1). After 2 h, the cells were washed again with PBS and treated with 150 μM of necrostatin-1 (Nec-1) or 0.05% DMSO in 10% DMEM (Con) for the indicated times. **(B)** Flow diagram of RD cells treated with caspase-3 inhibitor (Z-DEVD-FMK). RD cells were pre-treated with 20 μM of Z-DEVD-FMK inhibitor (Z-DEVD) or 0.05% DMSO in 10% DMEM (Con) for 2 h. The cells were then washed with PBS and mock-infected or infected with CA6 (MOI = 5) or EV71 (MOI = 1). After 2 h, the cells were washed again with PBS and re-treated with 20 μM of Z-DEVD-FMK or 0.05% DMSO in 10% DMEM for the indicated times. **(C)** Cell morphologic analysis with propidium iodide staining after CA6 (MOI = 5) infection was performed at 36 h post infection with necrostatin-1 or Z-DEVD-FMK treatment by light microscopy (left) or fluorescence microscopy (right). Results are representative of three independent experiments. Bar = 20 μm. **(D)** Cell morphologic analysis with propidium iodide staining after EV71 (MOI = 1) infection was performed at 24 h post infection with necrostatin-1 or Z-DEVD-FMK treatment by light microscopy (left) or fluorescence microscopy (right). Results are representative of three independent experiments. Bar = 20 μm. **(E)** Cell number analysis after CA6 infection (MOI = 5) at 36 h treatment with control, necrostatin-1, or Z-DEVD-FMK. The cell numbers were counted by trypan blue staining. The results represent the mean ± SD of three independent experiments. **P < 0.01. **(F)** Cell number analysis after EV71 infection (MOI = 1) at 24 h treatment with control, necrostatin-1, or Z-DEVD-FMK. The cell numbers were counted by trypan blue staining. The results represent the mean ± SD of three independent experiments. ***P < 0.001. Nec-1, necrostatin-1; Z-DEVD, Z-DEVD-FMK; Mock, Mock infection; Con, control.

To further evaluate the possibility that CA6 induces necroptosis, the necroptosis inhibitor, necrostatin-1 (150 μM, A4213, ApexBio, United States), and the apoptosis inhibitor, Z-DEVD-FMK (20 μM, A1920, ApexBio, United States), were added (Figures 2A,B). The results demonstrated that necrostatin-1 inhibited the morphological changes and PI permeability induced by CA6, although Z-DEVD-FMK had no effect (Figure 2C). On the other hand, Z-DEVD-FMK, but not necrostatin-1, inhibited the morphological changes induced by EV71 infection (Figure 2D). These results suggest that CA6 induces cell death by a mechanism that differs from the mechanism of apoptosis induced by EV71.

To verify that necrostain-1 modulates the cytopathic effect caused by CA6 virus, we examined the effect of necrostatin-1 on the cell number. Our results demonstrated that necrostatin-1 reversed the decrease in the cell number caused by CA6 infection (4.53 ± 0.06 × 10^5^ versus 3.33 ± 0.31 × 10^5^; *P* < 0.01), but Z-DEVD-FMK could not reverse the decrease induced by CA6 infection (Figure 2E). However, Z-DEVD-FMK reversed the decrease in the cell number caused by EV71 infection (3.90 ± 0.10 × 10^5^ versus 1.93 ± 0.15 × 10^5^; *P* < 0.001), but necrostatin-1 could not reverse this decrease induced by EV71 infection (Figure 2F). Therefore, these results are consistent with the ability of CA6 to induce necroptosis.

### RIPK3, a Protein in the Pathway of Necroptosis, Is Up-Regulated After CA6 Infection

**FIGURE 3:**
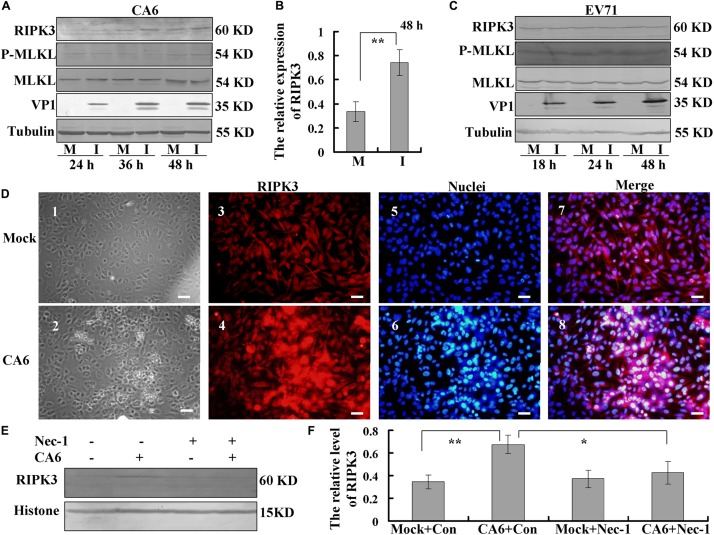
Analysis of host necroptosis regulatory proteins. **(A)** RD cells were mock-infected (M) or infected with CA6 at a MOI of 5 (I) and then collected at the indicated times. RIPK3, p-MLKL, MLKL, and VP1 were detected by Western blot analysis. Tubulin is shown as a loading control. Results are representative of three independent experiments. **(B)** RD cells were mock-infected (M) or infected with CA6 at a MOI of 5 (I) and then collected at the 48 h post infection. The band of RIPK 3 and corresponding tubulin was processed by ImageJ, and the ratio of intensity of RIPK3 to corresponding tubulin was shown. The results represent the mean ± SD of three independent experiments. **P < 0.01. **(C)** RD cells were mock-infected (M) or infected with EV71 at a MOI of 1 (I) and then collected at the indicated times. RIPK3, p-MLKL, MLKL, and VP1 were detected by Western blot analysis. Tubulin is shown as a loading control. Results are representative of three independent experiments. **(D)** RIPK3 distribution as assessed by fluorescence microscopy at 36 h post-infection with CA6 at a MOI of 5. Cells were stained with RIPK3 antibody, and then co-stained with CoraLite594-conjugated-second antibody (Red). DNA was counterstained with Hoechst 33258 (Blue). Cell morphology was visualized by light microscope. Merged images of RIPK3 with DNA are shown. Results are representative of three independent experiments. Scale bar = 20 μm. **(E)** Necrostatin-1 inhibited the expression of RIPK3-induced by CA6. Histone is shown as a loading control. Results are representative of three independent experiments. **(F)** The band of panel **(E)** was processed by ImageJ, and the ratio of intensity of RIPK3 to corresponding histone was shown. The results represent the mean ± SD of three independent experiments. **P < 0.01. *P < 0.05.

Next, we investigated the mechanism of necroptosis induced by CA6 infection by assessing the effects of CA6 infection on necroptosis proteins. The expression of RIPK3 was up-regulated by CA6 infection, with a similar time course of upregulation as that of the viral protein VP1, while the expression of P-MLKL and MLKL was not consistently altered (Figure 3A), and especially at 48 h post infection, the expression of RIPK3 was significantly up-regulated by CA6 infection comparing to mock infection (*P* < 0.01, Figure 3B); however, EV71 infection did not affect RIPK3 expression as well as the expression of P-MLKL and MLKL with time (Figure 3C), which proved that CA6 could regulate necroptotic pathway protein, but not EV71. Furthermore, the expression of the apoptotic proteins pro-caspase-3 and cleaved caspase-3 was not changed after CA6 infection (Supplementary Figure S1A), but was changed after EV71 infection (Supplementary Figure S1B), which further confirmed that CA6 infection did not induce apoptosis and was different from EV71. For further confirmation that RIPK3 is up-regulated by CA6 infection, we performed immunofluorescence analysis. The results confirm CA6 infection induced necroptosis (Figure 3D2) compared to mock infection (Figure 3D1), and the increased expression of RIPK3 was visualized in necroptotic cells induced by CA6-infection (Figure 3D4) compared to mock-infection (Figure 3D3). Furthermore, the up-regulated RIPK3 was also localized to the cytoplasm, where viral genome replication, protein translation, and viral package occur (Figure 3D8). We additionally assessed the effects of necrostatin-1 on RIPK3 expression. At 36 h post infection, the expression of RIPK3 underwent consistent decreased in response to necrostatin-1 treatment (*P* < 0.05, Figures 3E,F). Therefore, the necroptosis induced by CA6 infection is associated with the up-regulation of RIPK3.

### Necroptosis Promotes Viral Production

**FIGURE 4:**
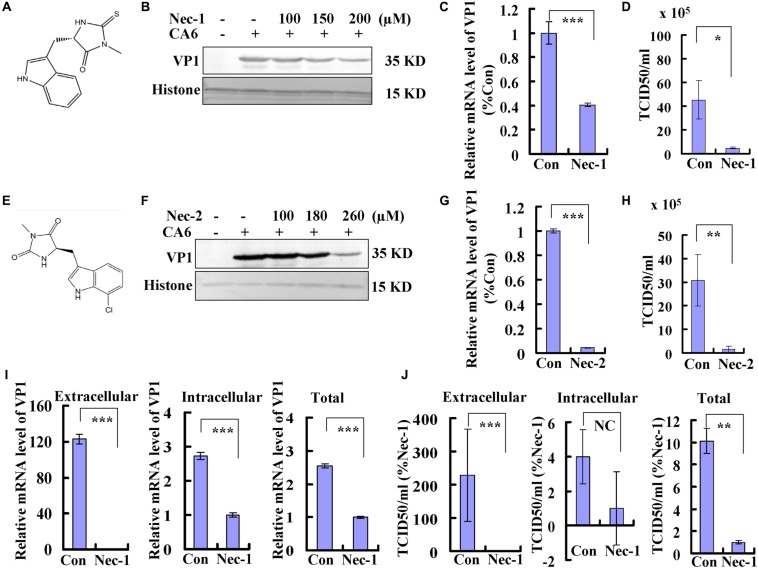
Necrostatin-1 or necrostatin-2 inhibits viral production. **(A)** The chemical structure of necrostatin-1. **(B)** VP1 expression in cellular lysates was determined after growth in control medium or different doses of necrostatin-1 medium at 36 h post-infection. The results are representative of three independent experiments. Histone is shown as loading control. **(C)** At 36 h post-infection, intracellular CA6 RNA levels were detected in control, necrostatin-1 (150 μM)-treated RD cells by real-time quantitative PCR. The results were standardized to GAPDH mRNA as a control and normalized to 1.0 in Con-treated cells. ***P < 0.001. **(D)** Total progeny viruses collected at 36 h post-infection of CA6 (MOI = 5) were titrated using RD cells. The results indicate the means ± SD of three independent experiments. *P < 0.05. **(E)** The chemical structure of necrostatin-2. **(F)** VP1 expression in cellular lysates was determined after growth in control medium or different doses of necrostatin-2 medium at 36 h post-infection. The results are representative of three independent experiments. Histone is shown as loading control. **(G)** At 36 h post-infection, intracellular CA6 RNA levels were detected in control, necrostatin-2 (180 μM)-treated RD cells by real-time quantitative PCR. The results were standardized to GAPDH mRNA as a control and normalized to 1.0 in Con-treated cells. ***P < 0.001. **(H)** Total progeny viruses collected at 36 h post-infection of CA6 (MOI = 5) were titrated using RD cells. The results indicate the means ± SD of three independent experiments. **P < 0.01. **(I)** At 36 h post-infection, extracellular, intracellular, and total CA6 RNA levels were detected in control, necrostatin-1 (150 μM)-treated RD cells by real-time quantitative PCR. The results of extracellular and intracellular were standardized to corresponding cell number and normalized to 1.0 in Nec-1-treated cells. ***P < 0.001. **(J)** At 36 h post-infection, extracellular, intracellular, and total virions were detected in control, necrostatin-1 (150 μM)-treated RD cells by TCID50. The results of extracellular and intracellular were standardized to corresponding cell number and normalized to 1.0 in Nec-1-treated cells. ***P < 0.001, **P < 0.01. NC, no significant difference; Con, control treatment; Nec-1, necrostatin-1; Nec-2, necrostatin-2.

Necrostatin-1 is (R)-5-([7-chloro-1H-indol-3-yl]methyl)-3-methylimidazolidine-2,4-dione (Figure 4A). Below the concentration of 200 μM, the inhibitory ratio of necrostatin-1 is <5%, the inhibitory ratio of 360 μM of necrostatin-1 is about 37% at 36 h post-treatment (Supplementary Figure S1C). To evaluate whether the necroptosis induced by CA6 infection affects viral production, we examined the effect of different doses of necrostatin-1 treatment (100, 150, and 200 μM) on viral protein expression at 36 h post infection. The expression of VP1 was decreased by necrostatin-1 in a dose-dependent manner (Figure 4B). To verify these findings, the effects of necrostatin-1 on CA6 genomic levels were evaluated. Necrostatin-1 obviously inhibited the intracellular viral genomic level as assessed by real-time PCR of the VP1 coding sequence (*P* < 0.001) (Figure 4C). Finally, we analyzed CA6 virus production at 36 h post-infection. The TCID50/mL of CA6-infected cells (45.27 ± 16.16 × 10^5^) was dramatically reduced by the addition of necrostatin-1 (4.66 ± 0.81 × 10^5^) (*P* < 0.05) (Figure 4D). At the same time, another necroptosis inhibitor, necrostatin-2, was utilized to confirm necroptosis affecting CA6 viral production. Necrostatin-2 is an analog of necrostatin-1 (Teng et al., 2007; Figure 4E) and has more powerful ability of inhibiting necroptosis and less cytotoxicity than necrostatin-1. Below the concentration of 280 μM, the inhibitory ratio of necrostatin-2 is <5%, the inhibitory ratio of 360 μM of necrostatin-2 is about 16% at 36 h post-treatment (Supplementary Figure S1D). And it was confirmed that necrostatin-2 had a similar inhibitory ability on viral production (Figures 4F–H). To further confirm the mechanism of necroptosis affecting viral production, we analyzed the effect of necrostatin-1 on viral release. Considering the alteration of cell number after virus infection and necrostain-1 treatment, before calculating intracellular and extracellular viral genome level and TCID50, the cell number was detected, and it was found that different dose of necrostatin-1 (Supplementary Figure S2A) and necrostatin-2 (Supplementary Figure S2B) could prevent the damage induced by CA6 infection, but not caspase-3 inhibitor (Supplementary Figure S2C). Especially, the survival ratio of CA6 infected cells is 66%, while together with 150 μM of necrostatin-1 the survival ratio of CA6 infected cells is 85% at 36 h post infection (Supplementary Figure S2A). Followed that, we detected the mRNA level of CA6 in extracellular, intracellular, and total samples by real-time PCR, then the relative extracellular and intracellular mRNA level was calculated assuming the same cell number in each group of control and necrostatin-1 treatment (150 μM), and it was found that excluding the effect of cell number, for extracellular mRNA level, control treatment was 123 times as large as the necrostatin-1 treatment, and for intracellular mRNA level, control treatment was 2.73-fold of the necrostation-1 treatment, while for total mRNA level, control treatment was 2.54 times than the necrostation-1 treatment (Figure 4I). Meanwhile, for extracellular virions production, control treatment was 229 times as large as the necrostatin-1 treatment, and for intracellular virions production, there was no obvious difference between control treatment and necrostation-1 treatment, while for total virions production, control treatment was 10 times than the necrostation-1 treatment (Figure 4J and Supplementary Figure S2D). Therefore, these results suggest that CA6-induced necroptosis promotes viral production, especially through inhibiting viral release.

### ROS Scavenger Do Not Inhibit CA6 Proliferation

**FIGURE 5:**
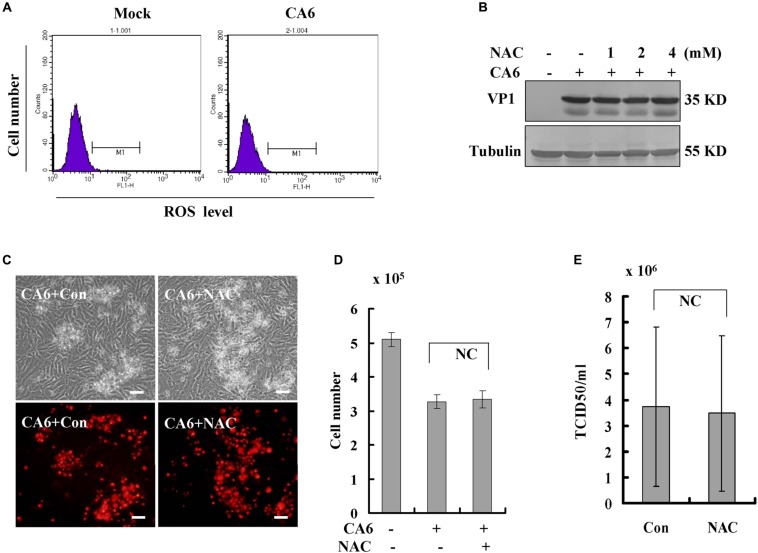
Scavengers of ROS (NAC) do not inhibit necroptosis or viral production. **(A)** The level of ROS was determined after CA6 infection (MOI = 5) at 36 h post-infection by flow cytometery. The results are representative of three independent experiments. **(B)** VP1 expression in cellular lysates was determined after growth in control medium or different doses of NAC medium at 36 h post-infection. The results are representative of three independent experiments. Tubulin is shown as loading control. The results are representative of three independent experiments. **(C)** Cell morphologic analysis with propidium iodide staining after CA6 (MOI = 5) infection was performed at 36 h post infection with control or NAC treatment (2 mM) by light microscopy (upper) or fluorescence microscopy (lower). Results are representative of three independent experiments. Bar = 20 μm. **(D)** Cell number analysis after CA6 infection at 36 h. The cell numbers were counted by trypan blue staining. The results represent the mean ± SD of three independent experiments. NC, no significant difference. **(E)** Total progeny viruses collected at 36 h post-infection of CA6 were titrated using RD cells. The results indicate the mean ± SD of three independent experiments. NC, no significant difference.

Often, necroptosis is associated with increases in the level of ROS (Takemoto et al., 2014; Schenk and Fulda, 2015; Muraro, 2018). To investigate the relationship between CA6 infection and ROS, the level of ROS was detected, CA6 infection did not change the level of ROS compared to the mock-infection (Figure 5A). To further confirm the relationship between CA6 infection and ROS, scavengers of ROS (NAC, *N*-Acetyl-L-cysteine, Sigma, St. Louis, MO, United States, A9165) were applied to CA6-infected cells. Increasing doses of NAC had no effect on the expression of VP1 (Figure 5B), the necroptotic death induced by CA6 (Figures 5C,D), or virion production (Figure 5E). To prove the effectiveness of ROS detection and NAC, pseudolaric acid (3 μM) was used as positive control (Qi et al., 2013), and it was found that pseudolaric acid increased the level of ROS (*P* < 0.001) and NAC inhibited the increase of ROS (*P* < 0.05) (Supplementary Figures S3A,B). Therefore, these results suggest that necroptosis induced by CA6 is not dependent on ROS.

### Viral Non-structural Protein 3D Directly Hijacks RIPK3

**FIGURE 6:**
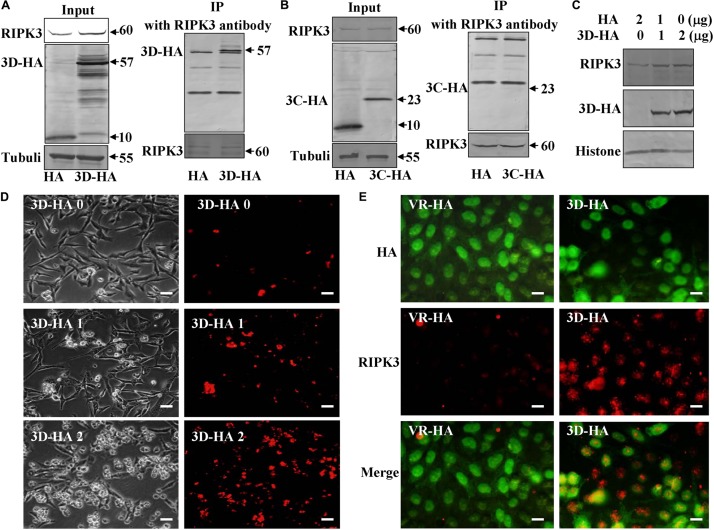
Non-structural protein 3D binds RIPK3. **(A)** Non-structural protein 3D in 3D-transfected cells was evaluated by Western blotting before or after immunoprecipitation (IP) with RIPK3 antibody (Santa) at 36 h post transfection. Results are representative of three independent experiments. **(B)** Non-structural protein 3C in 3C-transfected cells was evaluated by Western blotting before or after immunoprecipitation (IP) with RIPK3 antibody (Santa) at 36 h post transfection. Results are representative of three independent experiments. **(C)** The expression of RIPK3 in 293T cells was analyzed at 36 h after transfection with 0, 1, or 2 μg of 3D-HA-VR1012 plasmid as indicated. Results are representative of three independent experiments. Histone is loading control. **(D)** Transfection of 0, 1, or 2 μg of 3D-HA-VR1012 plasmid into RD cells (HA-VR1012 plasmid was used to replenish 2 μg/well) for 36 h, cell morphologic analysis with propidium iodide staining by light microscopy (left) or fluorescence microscopy (right). Results are representative of three independent experiments. Bar = 20 μm. **(E)** The localization of RIPK3 and HA was analyzed by fluorescence microscopy at 48 h post-transfection of VR1012-HA and VR1012-3D-HA. Cells were fixed in 4% paraformaldehyde, stained with RIPK3 and HA antibodies, and then co-stained FITC labeled-secondary antibody (Green, HA) and CoraLite594-conjugated-second antibody (Red, RIPK3). Merged images of HA and RIPK3 are shown. Data are representative of three individual experiments. Scale bar = 10 μm.

As an alternative to ROS induction, we speculated that CA6 virus might directly hijack the pathway of necroptosis by targeting RIPK3 protein. Viral non-structural protein 3C as a protease and 3D as an RNA-dependent RNA polymerase play important role in viral life cycle (Song et al., 2018; Wang Z. et al., 2018), and 3C and 3D of CA6 induce G0/G1 cell cycle arrest (Wang Z. et al., 2018). Therefore, we firstly evaluated the interaction between these proteins by immunoprecipitation assay, RIPK3 protein antibody was able to pull down 3D-HA but not 10 KD of HA which was constructed by our previous study (Song et al., 2018; Figure 6A). However, RIPK3 protein antibody was not able to pull down 3C-HA (Figure 6B). Therefore, RIPK3 possibly was hijacked by viral 3D protein but not 3C protein. In the following study, it was found that with the increase of 3D protein, the expression of RIPK3 was correspondingly up-regulated (Figure 6C), and with the 3D increase, necroptosis occurred more and more (Figure 6D). We further evaluated that RIPK3 can co-localize with the viral non-structural 3D. The results demonstrate that RIPK3 was up-regulated by 3D-HA and co-localized with 3D-HA but not HA (Figure 6E). On the basis of these results, 3D protein may regulate necroptosis by binding RIPK3.

## Discussion

Apoptosis is a prevalent form of programmed cell death that is activated by many viruses, such as CA16 (Li et al., 2014), human immunodeficiency virus (HIV-1) (Westendorp et al., 1995), hepatitis C virus (Zhu et al., 1998), Epstein–Barr virus (Le Clorennec et al., 2006), and human papillomavirus (Wang et al., 2011). Previous studies show that EV71, which is a major pathogen of typical HFMD, induces apoptosis (Chang et al., 2004) and activates caspase-3 for own production (Song et al., 2018). However, when we investigated the death mechanism of the CA6 virus, the major causative agent of atypical HFMD, we found that CA6 does not induce the same program of apoptosis as EV71.

Apoptotic cells display characteristic morphological features, including membrane blebbing, condensed cells, and the formation of apoptotic bodies, which can be observed after EV71 infection. However, our results reveal that after CA6 infection, membrane rupture and organelle swelling occur, which are the hallmarks of necroptosis. Furthermore, the cell death induced by CA6 infection was attenuated by necrostatin-1, but not caspase-3 inhibitor. Therefore, our results suggest that CA6 induces necroptosis rather than apoptosis.

The induction of necroptosis by CA6 virus could potentially explain the atypical manifestation of HFMD. HFMD is a febrile exanthematous disease with typical or atypical symptoms in children younger than 5 years of age. EV71 infection induces typical symptoms of HFMD, including flat discolored spots or bumps, even heart inflammation, brain inflammation, or acute flaccid paralysis (Chan et al., 2003; Wang et al., 2012), while CA6 leads to atypical HFMD characterized by severe rash, onychomadesis in young children, and a higher rate of infection in adults. In recent years, the large outbreaks of HFMD are usually caused by EV71 infection, while CA6-associated HFMD has lower rates that have markedly increased worldwide. Therefore, apoptosis or necroptosis might be related to varying clinical characteristics, such as the epidemic region, epidemic frequency, clinical symptoms, and epidemic size.

Although CA6 diverges from the typical HFMD viruses in its ability to promote necroptosis, there are many other viruses that activate necroptosis. BPV (Abdel-Latif et al., 2006), murine cytomegalovirus (Upton et al., 2012), influenza A virus (Thapa et al., 2016), and reovirus (Berger et al., 2017) are among numerous other viruses that have this ability (Upton et al., 2012; Nogusa et al., 2016; Berger et al., 2017; Maelfait, 2017; Schock et al., 2017; Guo et al., 2018; Muraro, 2018; Gaba et al., 2019; Lee et al., 2019). Furthermore, some viruses, such as Epstein–Barr virus (Liu et al., 2018) and human cytomegalovirus (Omoto et al., 2015), can inhibit necroptosis. Therefore, the regulation of necroptosis is a common theme among a subset of viruses, and our results provide a new model to investigate mechanisms associated with necroptosis regulation.

The core of the necroptotic pathway involves the activities of the RIP kinases, RIPK1 and RIPK3, and their ability to phosphorylate and activate the pseudokinase mixed lineage kinase domain-like (MLKL) protein. We demonstrated that RIPK3 is up-regulated after CA6 infection and that the up-regulation of RIPK3 is reversed by the necroptosis inhibitor necrostatin-1, suggesting that RIPK3 may mediate CA6-induced necroptosis. However, the expression of MLKL and p-MLKL was not affected by CA6 infection. Therefore, our results are consistent with the possibility that RIPK3 plays a role in CA6-induced necroptosis that is independent of its ability to phosphorylate MLKL. Future studies to identify downstream effectors of RIPK3 activation in CA6-infected cells may address this possibility.

Necroptosis induced by viral infection has been shown to inhibit viral proliferation (Nogusa et al., 2016; Guo et al., 2018; Lee et al., 2019) or to promote viral magnification (Bozym et al., 2011), depending on the virus and the context. In this study, we demonstrated that necrostatin-1 inhibits viral production. These results were verified by measurement of VP1 genomic levels and protein expression and by assessing the TCID50. In the further study, it was found that necrostatin-1 decreased the extracellular (123-fold), intracellular (2.73-fold), and total (2.54-fold) viral mRNA level, decreased extracellular (229-fold) and total (10-fold) virions, which indicated that necrostatin-1 inhibited virus production, especially viral release. Therefore, our results suggest that necroptosis is beneficial to the CA6 virus, especially viral release, although further analysis will be needed to elucidate the exact mechanism.

It has been reported that necroptosis is associated with the generation of ROS (Muraro, 2018). However, in our study, we found that CA6 infection did not increase the ROS level, and scavengers of ROS did not affect the necroptosis induced by CA6 or the production of CA6. As an alternate mechanism, viral 3D protein was shown to bind RIPK3 protein, but not viral 3C protein. Further study confirmed that 3D protein up-regulated the expression of RIPK3 and induced necroptosis. Therefore, it is speculated that the virus might directly hijack a factor related to necroptosis to induce necroptosis, which provides a mechanism to explain CA6 induction of necroptosis.

## Conclusion

In conclusion, necroptosis is exploited functionally by CA6 to facilitate its production, which suggests a novel therapeutic approach for the treatment and prevention of HFMD.

## Data Availability Statement

All datasets generated for this study are included in the article/Supplementary Material.

## Author Contributions

JY designed the experiments and wrote the manuscript. JY, SZ, and XY analyzed the results. SZ, XM, WH, YS, JL, YL, and JZ conducted the experiments. JY, SZ, XY, XM, WH, YS, JL, YL, JZ, and SW prepared the viruses, the cell lines, and the reagents, and discussed the data.

## Conflict of Interest

The authors declare that the research was conducted in the absence of any commercial or financial relationships that could be construed as a potential conflict of interest.

## Footnotes

1 https://www.ncbi.nlm.nih.gov/nuccore/1071451807
