# Supplementary material for: Coxsackievirus A6 Induces Necroptosis for Viral Production
Source: Front Microbiol. 2020 Feb 4;11:42. doi: 10.3389/fmicb.2020.00042 (PMC7011610; doi:10.3389/fmicb.2020.00042)
Supplement: Supplementary file 1 [file Table_1.DOC]

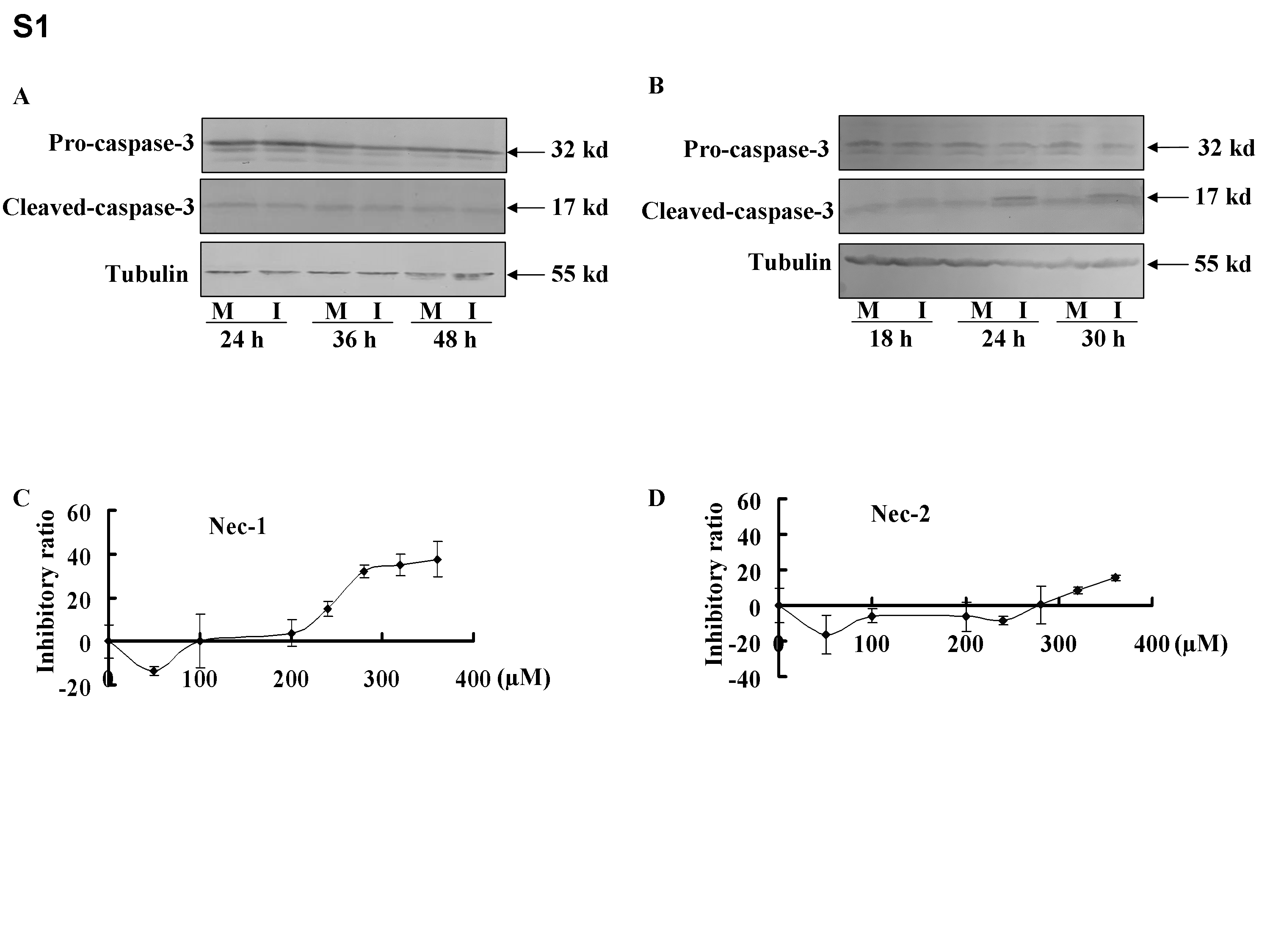


S1. The expression of pro-caspase-3 and caspase-3 after CA6 infection. (A) RD cells were mock-infected (M) or infected with CA6 at an MOI of 5 (I) and then collected at the indicated times for pro-caspase-3 and caspase-3 detected by Western blot analysis. Tubulin was shown as a loading control. Results were representative of three independent experiments. (B) The expression of pro-caspase-3 and caspase-3 after EV71 infection. RD cells were mock-infected (M) or infected with EV71 at an MOI of 1 (I) and then collected at the indicated times for pro-caspase-3 and caspase-3 detected by Western blot analysis. Tubulin was shown as a loading control. Results were representative of three independent experiments. (C) The cells were treated with different dose of necrostatin-1 (Nec-1, 0 μM, 50 μM, 100 μM, 200 μM, 240 μM, 280 μM, 320 μM and 360 μM) for 36 h. Inhibition of cell growth was determined by MTT (Sigma, St Louis, MO, USA) assay. Absorbance was measured at 492 nm with an enzyme-linked immunosorbent assay plate (ELISA) reader (Bio-Rad, Hercules, CA, USA). The percentage of inhibition was calculated as follows: Inhibitory ratio (%)=[A492(control)–A492(sample)]/[A492(control)–A492(blank)]×100%. (D) The cells were treated with different dose of necrostatin-2 (Nec-2, 0 μM, 50 μM, 100 μM, 200 μM, 240 μM, 280 μM, 320 μM and 360 μM) for 36 h. Inhibition of cell growth was determined by MTT (Sigma, St Louis, MO, USA) assay. Absorbance was measured at 492 nm with an enzyme-linked immunosorbent assay plate (ELISA) reader (Bio-Rad, Hercules, CA, USA). The percentage of inhibition was calculated as follows:Inhibitory ratio (%)=[A492(control)–A492(sample)]/[A492(control)–A492(blank)]×100%.


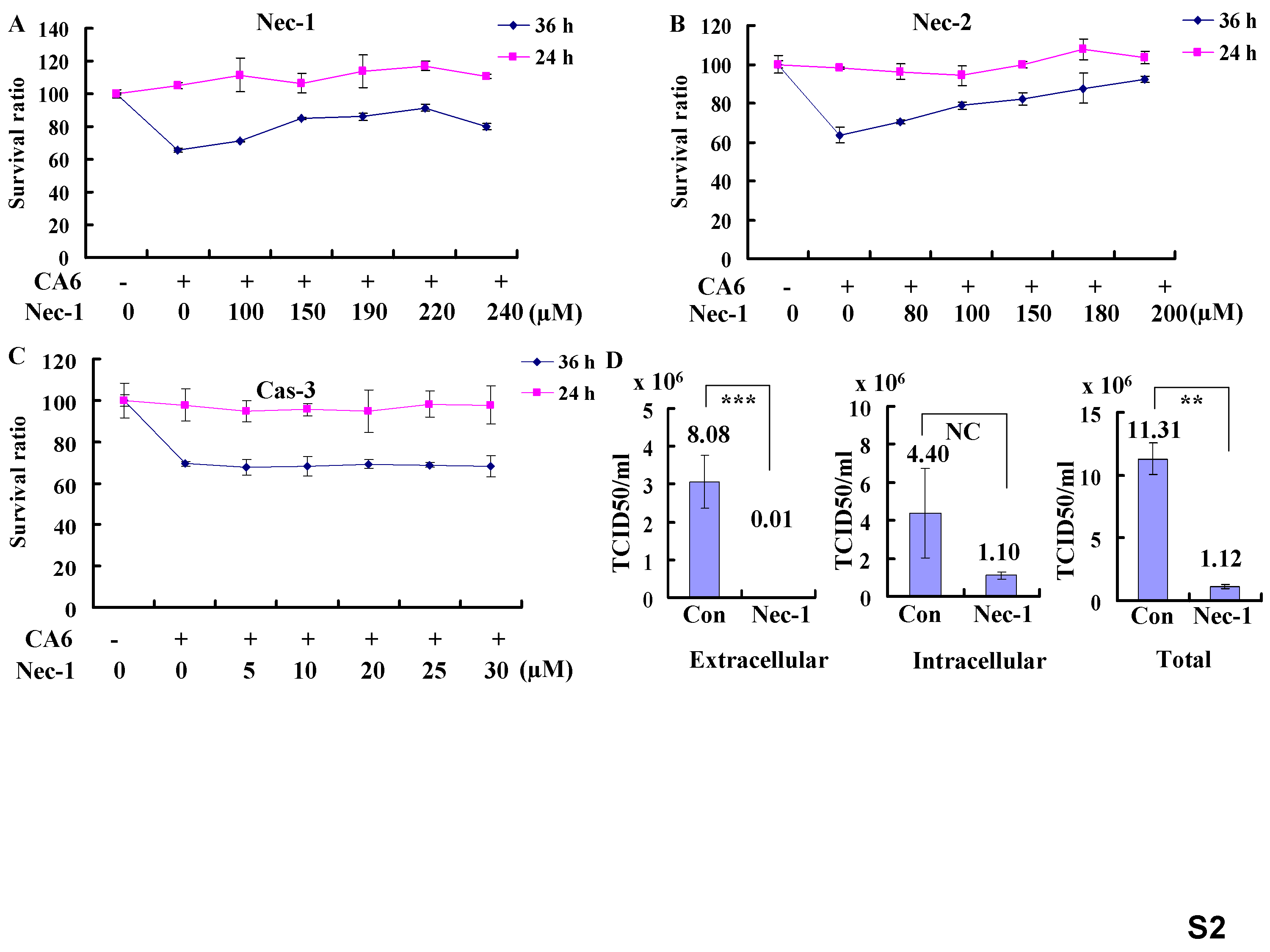


S2. The protective role of necrostatin-1 and necrostatin-2 on cell death induced by CA6 infection. (A) RD cells were infected with CA6 at an MOI of 5, after 2 h, the cells were washed again with PBS and treated with 0 μM, 100 μM, 150 μM, 190 μM, 220 μM and 240 μM necrostatin-1 (Nec-1) for 24 h and 36 h. Absorbance by MTT (Sigma, St Louis, MO, USA) assay was measured at 492 nm with an enzyme-linked immunosorbent assay plate (ELISA) reader (Bio-Rad, Hercules, CA, USA). The survival ratio was calculated as follows: A492(sample)/[A492(control)–A492(blank)]×100%. (B) RD cells were infected with CA6 at an MOI of 5, after 2 h, the cells were washed again with PBS and treated with 0 μM, 80 μM, 100 μM, 150 μM, 180 μM and 200 μM necrostatin-2 (Nec-2) for 24 h and 36 h. Absorbance by MTT (Sigma, St Louis, MO, USA) assay was measured. (C) RD cells were pre-treated with 5 μM, 10 μM, 20 μM, 25 μM and 30 μM of Z-DEVD-FMK inhibitor (Cas-3) for 2 h. The cells were then washed with PBS and infected with CA6 (MOI=5). After 2 h, the cells were washed again with PBS and re-treated with different dose of Z-DEVD-FMK for 24 h and 36 h. Absorbance by MTT (Sigma, St Louis, MO, USA) assay was measured. (D) At 36 h post-infection, extracellular, intracellular and total virions were detected in control or necrostatin-1 (150 μM) treated RD cells by TCID50. The results of extracellular and intracellular were standardized to corresponding cell number. ***P < 0.001, **P < 0.01, NC: no significant difference. Con: control treatment; Nec-1: necrostatin-1; Nec-2: necrostatin-2.


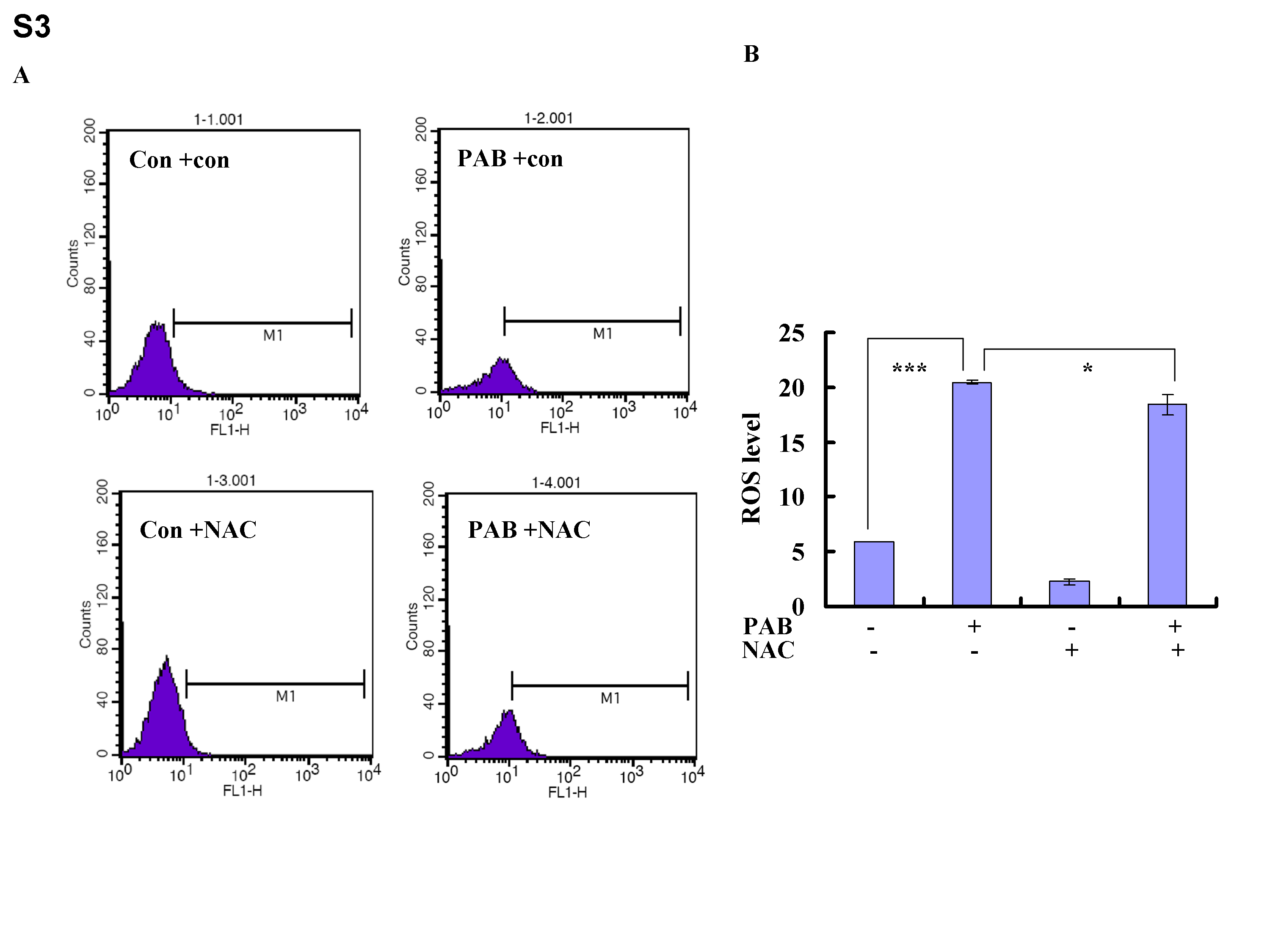


S3. Pseudolaric acid increased the level of ROS. (A) The level of ROS was determined after Pseudolaric acid treatment (3 μM) together with NAC treatment (2 mM) at 30 h post-treatment by flowcytometery. The results are representative of three independent experiments. (B) The histograms show the level of ROS.
